# An Overview of the History, Pathophysiology, and Pharmacological Interventions of Multiple Sclerosis

**DOI:** 10.7759/cureus.33242

**Published:** 2023-01-02

**Authors:** Ibrahim M Dighriri, Ahood A Aldalbahi, Fatimah Albeladi, Asimah A Tahiri, Elaf M Kinani, Rand A Almohsen, Nouf H Alamoudi, Abeer A Alanazi, Sultan J Alkhamshi, Noha A Althomali, Sultan N Alrubaiei, Faisal K Altowairqi

**Affiliations:** 1 Department of Pharmacy, King Abdulaziz Specialist Hospital, Taif, SAU; 2 Department of Medicine, PHC Al-Qassim Health Cluster, Buraidah, SAU; 3 Department of Pharmacy, Maternity and Children Hospital, Dammam, SAU; 4 Department of Pharmacy, Armed Forces Hospital, Jazan, SAU; 5 Department of Pharmacy, Al Thaghr Hospital, Jeddah, SAU; 6 Faculty of Pharmacy, University of Shaqra, Al-Dawadmi, SAU; 7 Faculty of Pharmacy, Umm Al Qura University, Mecca, SAU; 8 Faculty of Pharmacy, Northern Border University, Rafha, SAU; 9 Faculty of Pharmacy, Buraydah College, Al-Qassim, SAU; 10 Department of Pharmacy, Taif University, Taif, SAU; 11 Department of Pharmacy, Community Pharmacy, Riyadh, SAU

**Keywords:** cns, pathogenesis, disease-modifying therapies, ms, multiple sclerosis

## Abstract

Multiple sclerosis (MS) is an immune-inflammatory disease that attacks and damages myelinated axons in the central nervous system (CNS) and causes nontraumatic neurological impairment in young people. Historically, Lidwina of Schiedam documented the first MS case. After that, Augustus d'Este wrote for years about how his MS symptoms worsened. Age, sex, genetics, environment, smoking, injuries, and infections, including herpes simplex and rabies, are risk factors for MS. According to epidemiology, the average age of onset is between 20 and 40 years. MS is more prevalent in women and is common in Europe and America. As diagnostic methods and criteria change, people with MS may be discovered at earlier and earlier stages of the disease. MS therapy has advanced dramatically due to breakthroughs in our knowledge of the disease's etiology and progression. Therefore, the efficacy and risk of treatment medications increased exponentially. Management goals include reducing lesion activity and avoiding secondary progression. Current treatment approaches focus on managing acute episodes, relieving symptoms, and reducing biological activity. Disease-modifying drugs such as fingolimod, interferon-beta, natalizumab, and dimethyl fumarate are the most widely used treatments for MS. For proof of the efficacy and safety of these medications, investigations in the real world are necessary.

## Introduction and background

Multiple sclerosis (MS) is an immune inflammatory disease that assaults myelinated axons in the central nervous system (CNS), damaging the myelin and axon to varying degrees [1]. Inflammation, neurodegeneration, and gliosis are hallmarks of MS. Perivascular lymphocytic infiltrate and macrophages destroy the myelin sheaths that pathologically wrap neurons [2,3]. In most cases, the condition is relapsing-remitting, with brief bouts of neurologic impairment that resolve entirely or nearly wholly [4]. MS pathophysiology is not fully understood but may be connected to hereditary predisposition and a putative non-genetic trigger that results in a sustaining autoimmune disease leading to recurring immunological attacks on CNS [5]. The geographic variance in the incidence of MS suggests environmental causes [3,5]. Due to improvements to the criteria used to make these assessments, people may now get a more accurate and quick diagnosis [4].

Recent breakthroughs in the knowledge of MS processes have led to the development of innovative therapy methods [5,6]. Recently, numerous drugs have been effective in phase III testing, indicating approval [7]. As viable therapy increases, choosing the correct one for each patient is harder. Modern medicine treats this condition with hormones, immunosuppressants, plasma exchange, and other medications [8]. As a clinically incurable disease, MS burdens patients and communities [9]. As a result, how to manage MS safely and efficiently has become a pressing societal medical issue [3,10]. Therefore, this study aims to review the history, pathophysiology, and pharmacological interventions of MS.

## Review

History

The term "paraplegia" refers to any severe neurological disorder marked by motor impairment. Saint Lidwina of Schiedam, who lived in the Netherlands at the end of the 14th century, documented the first mention of MS [11,12]. Augustus d'Este kept a journal for 26 years in which he wrote about how symptoms of what we now know to be MS got worse over time. His initial sign was a temporary visual impairment, most likely optic neuritis, at age 28. He died at age 54 of motor symptoms and lower extremities that hindered his walking [11-14]. Charcot's naming and framing of MS provided a framework for organizing previously unexplained discoveries and making future advances in MS. Since then, the consolidation has continued. Charcot's students correlated the disease's clinical symptoms with postmortem lesion pathology. Joseph Babinski's 1885 MS thesis described plaques in the brain and spinal cord. Pierre Marie highlighted autonomic dysfunction and gait impairments in MS [11,15]. Ernst Leyden initially hypothesized a hereditary component of MS in the mid-19th century. Still, it wasn't until the 1930s that Curtius and others in Germany started systematically assessing the genetics of MS and how the illness clustered in certain families [11,13,14].

Charcot, Von Frerichs, Vulpian, and others differentiated and "framed" MS as a unique, recognizable entity [12,15]. MS cases were classified according to their histology, clinical appearance, and prognosis before being diagnosed by doctors worldwide [13,14]. As awareness of MS developed, so made etiology ideas and therapeutic attempts; a 1935 research study included 158 MS treatments. Subsequent "cures" included anticoagulants, histamine desensitization, various diets, immunizations, and anticancer drugs [13,16]. Randomized clinical trials became more precisely defined in the decades following the 1960s, aided by advances in sickness classification and disability indices. Hypotheses were developed to account for immunological changes, genetic influences, geographical differences, infections, and environmental factors [6]. MS organizations enhanced research and general education and altered attitudes regarding the condition [16,17]. Last years, significant advances have been made in fundamental analysis to clarify the disease's causes and processes and immunomodulatory medications [11,16,17].

Etiology

Several risk factors lead to MS's development, including age, sex, race, heredity, geography, and infections such as herpes simplex, chlamydia, and rabies [18,19]. MS is likely the result of a complicated interplay between genetics, food, and the environment [6,20]. MS is primarily caused by an autoimmune attack on the CNS due to hyper immunity. Numerous postulated pathways have been proposed, but the proposed "outside-in" mechanism involves CD4+ proinflammatory T cells [21]. Researchers hypothesize that an unknown antigen promotes and activates 1 T-helper (Th1) and 17 T-helper (Th17), leading to CNS endothelium adhesion, blood-brain barrier (BBB) crossing, and subsequent immune attack through cross-reactivity. The "inside-out" theory posits that an innate malfunction of CNS produces and culminates in inflammation-mediated tissue destruction [21]. The phenomenon of environmental impacts, such as latitudinal gradients in different countries, has been widely studied [22]. A deficiency in vitamin D has been a possible explanation for susceptibility observed in populations living at higher latitudes [23,24]. Individuals with relatives have a considerable chance of developing the disease. The expected range of heritability is between 35 and 75% [25,26]. Human leukocyte antigen DRB1*1501 has a significant association with MS [27].

Risk factors

Vitamin D Deficiency

Vitamin D's activity in lymphocyte stimulation and modulation of growth and immune response suggests that it plays a substantial role in the pathogenesis of MS [19]. In addition, the reactions of the innate immune system and adaptive immunological activity are increased. Vitamin D reduces the production of Th1-mediated proinflammatory cytokines [28]. In many trials, vitamin D administration dramatically altered interleukin-10 (IL-10) and interleukin-17 (IL-17) levels [29,30]. MS is more common in people living farther north or south of the equator. The prevalence rate in societies near the equator is almost non-existent but grows to 50 cases per 1,000,000 individuals living 45 degrees north or south. Vitamin D insufficiency among MS patients is likely a contributor to this fascinating regional distribution [1,20,31,32].

Genetics and Family History

There is proof that some individuals have an inherited susceptibility to MS. However, this genetic susceptibility is not inherited since there is no MS-specific gene [33,34]. Genetic studies have shown a connection between first, second, and third-degree relatives [35,36].

Diseases

It has been postulated that bacterial or viral infections may promote the later development of MS in genetically susceptible individuals. Disorders in late childhood may introduce foreign antigens that activate Th1 cells and induce the characteristic of the autoimmune response of MS [20,31].

Injury

Severe injuries that directly damage the brain or spinal cord have been investigated as possible triggers for MS. Trauma increases the permeability of the BBB, thus facilitating the entry of Th1 cells into the CNS. This is the initiating factor for the inflammatory response that leads to myelin destruction and the formation of MS lesions [37,38].

Cigarette Smoking

Smoking is associated with higher risk of MS. Smokers with MS have a worse long-term prognosis and a greater incidence of brain atrophy than non-smokers [39]. In addition, MS sufferers are more likely to smoke than general population [40]. MS patients are more likely than the general population to have comorbid conditions associated with worse quality of life, a higher burden of disability, and higher mortality rates [41,42].

Epidemiology

MS is one of the most prevalent neurologic illnesses in the world and, in many countries, the leading cause of non-traumatic neurologic impairment in young people [43]. MS affects around 400,000 people in the United States and 2.5 million worldwide [44]. MS is now more prevalent among women; however, this was not always the case. In the early 1900s, the sex ratio was about equal. Since then, the sex ratio in most industrialized nations has progressively increased and is close to 3:1 (F: M) [44,45]. Smoking raises MS risk by about 50% [46]. Although the average age of onset is between 20 and 40 years, the disease can manifest at any age. Almost 10% of cases are diagnosed before the age of 18. In some cases, MS diagnosed after the age of 50 is known as "late-onset multiple sclerosis" (LOMS), which is a rare case [47]. Populations of European ancestry are estimated to have a prevalence of one in 1000. Less is known about prevalence among non-European groups, and most research points to a lower frequency among individuals of East Asian and African heritage. Recent research has shown that African-American communities have a prevalence rate comparable to European communities [48,49]. MS has a prevalence gradient dependent on latitude, with a higher incidence in the northern latitudes of Europe and North America. Observations indicating varied genetic susceptibility factors have also been observed in distinct human subpopulations, independent of latitude, indicating the interaction of poorly understood genetic and environmental components. Multiple studies have shown that populations that relocate to regions with a higher incidence of MS during infancy have a higher chance of developing the disease [50,51].

Pathophysiology

MS refers to the formation of plaques in CNS along with inflammation, demyelination, axonal damage, and axonal loss. These plaques are located in the brain and spinal cord, mainly in the white matter surrounding the ventricles, optic nerves and tracts, corpus callosum, cerebellar peduncles, long tracts, and subpial area of the spinal cord and brainstem, as well as gray matter. They have expressed in all forms of MS (primary, secondary, and relapsing-remitting MS). Still, their expression varies over time, demonstrating a profound heterogeneity in the immunopathological patterns of demyelination and oligodendrocyte degeneration between the relapsing-remitting course and the progressive forms of the disease [52,53]. MS is considered an autoimmune disease caused by autoreactive immune cells that traverse BBB and attack the CNS. Regular deletion of autoreactive immune cells during development occurs in the thymus or bone marrow through central tolerance B cells. Although some may escape this process and be released into circulation, peripheral tolerance mechanisms prevent them from causing disease in most cases. The impaired function of regulatory T cells and the resistance of autoreactive T cells to suppression are two mechanisms through which peripheral tolerance might fail. A complicated interaction between genetic and environmental risk factors may affect the activity and activation of these autoreactive cells, therefore contributing to the development of disease [52,54,55]. The primary T cell subsets implicated in MS include CD8+ T cells, CD4+ Th1 cells, and Th17 cells. Interferon-gamma, IL-17, and granulocyte-macrophage colony-stimulating factors are cytokines produced by autoreactive T cells that may contribute to the pathophysiology of MS [54]. The increased immunoglobulin in cerebral fluid suggests a role for B cells in MS. Intrathecal production of oligoclonal immunoglobulins, also known as oligoclonal bands (OCBs), is a diagnostic feature of MS. In MS, the majority of B cells in cerebrospinal fluid (CSF) and brain parenchyma are CD27+ memory B cells. Memory B cells are clonally enlarged in the CSF and brain parenchyma and exhibit somatic hypermutation and class-switched immunoglobulin transcripts. Furthermore, the overlap of the CSF immunoglobulin proteomes and the B cell immunoglobulin transcriptomes provides evidence that antibody-secreting cells derived from clonally expanded B cells within the CNS are a significant source of excessive intrathecal clonal immunoglobulin production, as demonstrated by the presence of OCBs in CSF [54,56].

Meninges in MS patients include inflammatory B cell infiltrates, and a greater load of these injects to the severity of cortical lesions, neurodegeneration, and clinical impairment. B cells may serve as Epstein-Barr Virus (EBV) reservoirs [53,54]. After EBV infection, B cells transform into antigen-processing cells, resulting in a more precise presentation of antigens. Recombinant human myelin oligodendrocyte glycoprotein was shown to be internalized and cross-presented by EBV-infected B-cells, which were efficiently identified by cytotoxic CD8+ T-cells. Furthermore, B cells obtained from MS patients had more CD40 on their surface, indicating that B cells deliver antigens more effectively [24]. Increased expression of B cell activation markers in individuals with relapsing-remitting multiple sclerosis (RRMS) was related to a high degree of neurodegeneration, as indicated by a rise in the number of T1 hyperintense lesions and a decrease in brain volume. In addition to B-cell-related diseases, loss of normal functioning in the effector T-cell population can contribute to the course of MS [24]. In healthy people, CD8+ cytotoxic T cells that eliminate EBV-infected lymphoblastoid cell lines keep EBV infection under control. Since particular cytotoxic CD8+ cells are prepared to identify and kill infected cells that express EBV latent proteins, they will be referred to as "latency-specific T cells" from now on. During MS exacerbation, the EBV-specific T-cell population expands, and the latency-specific CD8+ T-cell activity increases. However, as MS progresses, latency-specific CD8+ T-cells exhibit a fatigued phenotype and cannot inhibit the proliferation of latently infected cells. This results in a vicious loop in which an increased number of infected cells inhibits the autoregulatory system and further depletes T cells. Recurrent relapses can be linked to poor management of EBV reactivation, leading to increased infection of naive B cells and viral generation [24].

Antigen presentation to T cells and releasing chemicals that may harm oligodendrocytes are additional pathogenic pathways involving B cells in MS [54]. Microglia and macrophages release many cytokines, including tumor necrosis factor (TNF)-α, and interleukin (IL)-1β, which can contribute to neurodegeneration through cytokine-induced cell death, inhibition of astrocytic glutamate reuptake, and induction of dysfunctional ribonucleic acid-binding proteins. Microglia and macrophages can also release glutamate, which might contribute to glutamate excitotoxicity and neurodegeneration. Microglia and macrophages produce reactive oxygen/nitrogen species, which can contribute to dementia by generating oxidative stress and mitochondrial damage. Microglia can also express anti-inflammatory phenotypes, promoting remyelination [54].

Clinical presentation

When a patient arrives with a clinically isolated condition, MS is often assumed. Depending on the location of the eloquent lesion, it could be uni- or multisymptomatic. Brainstem, spinal cord syndrome, and optic neuritis are the most common presentations; nevertheless, there are various other, less typical presentations, including cortical presentations such as dominant parietal lobe syndromes [57]. Relapses of MS often develop subacutely over hours or days, plateau for many weeks, and then recover gradually. In early MS, gross clinical recovery after relapse typically looks complete; however, most relapses leave behind damage [58]. For instance, gross visual acuity may improve after acute optic neuritis, but impairments in color vision, contrast sensitivity, and depth perception remain. As the neuronal reserve is depleted, recovery from relapses becomes insufficient, and neuron deficiencies accumulate, resulting in permanent impairment [59]. On magnetic resonance imaging (MRI), approximately 10 "asymptomatic" lesions are seen for each clinical attack. A minor lesion in the eloquent region is likely to produce symptoms. Macroscopic or MRI-visible lesions are only the tip of the iceberg; many more lesions are apparent at the microscopic level, and many more are in deep and cortical gray matter. Secondary progressive MS occurs approximately 10-15 years after the beginning of RRMS, with a steady progression from isolated relapses to a slowly progressing disease. There is no clear transition between the disease categories; relapses occur against a backdrop of gradual progression until passage becomes predominant [59]. Cognitive impairment and an increase in MRI atrophy in early MS imply neurodegeneration from the outset of clinical symptoms. In 5 to 15% of cases, primary progressive multiple sclerosis (PPMS) is characterized by the slow accumulation of progressive impairment that affects the dominant neural system [57]. Progressive spastic paraparesis is the most prevalent manifestation of PPMS, although sensory ataxia, cerebellar ataxia, cognitive impairment, and advanced visual failure are well-described variations. There has been a decline in the percentage of individuals with PPMS [57,58].

 Diagnosis

McDonald's criteria are widely used in both clinical and research settings. In light of scientific advancements over the past seven years, these guidelines may no longer offer the most current information for physicians and researchers [60,61]. MS Diagnosis examined the McDonald criteria and suggested modifications [60]. McDonald's standards in 2017 continue to apply primarily to patients with a specific clinically isolated disease, define what is necessary to fulfill the spread in time and space of CNS lesions, and highlight that there must be no alternative explanation for the presentation [60,61] (Table 1).

**Table 1:** MS diagnosis according to McDonald's criteria MS: multiple sclerosis, CSF: cerebrospinal fluid, CNS: central nervous system, MRI: magnetic resonance imaging, OCBs: oligoclonal bands [60,61]

McDonald criteria	
Clinical presentation	Additional information required
Attacks: ≥ 2 Clinical evidence ≥ 2 lesions with historical evidence of past attack.	None. Clinical evidence is adequate. Further evidence is desirable.
Attacks: ≥ 2. Clinical evidence of one lesion.	Transmission in space as shown by MRI, or waiting for additional clinical research involving a different site.
Attacks: 1. Clinical evidence ≥ 2 lesions.	Timing of dissemination exhibited by MRI *or *second attack or demonstration of OCBs in the CSF.
Attacks: 1. Clinical evidence of one lesion.	Space dissemination is demonstrated by MRI or waiting for a second attack implicating a different CNS site. and time dissemination confirmed via MRI or a second attack.
Insidious neurologic progression is indicative of MS	Year of disease development and spread in space, demonstrated by1 or more T2 lesions in the brain in areas characterized by MS 2 or more T2 spinal cord focal lesions with positive CSF.

Management and treatment

Current therapy options focus on treating acute episodes, alleviating symptoms, and decreasing biological activity. The predominant therapy for MS is disease-modifying medications such as dimethyl fumarate, interferon-beta, natalizumab, and fingolimod. When the diagnosis of MS is obtained, immediate treatment should proceed. Short-term objectives include reducing MRI lesion activity. Long-term goals involve preventing secondary progressive MS. After beginning the medication, patient compliance and drug toxicity monitoring are the key concerns [3,62].

Ocrelizumab selectively depletes CD20-expressing B cells while retaining preexisting humoral immunity and the ability to reconstitute B cells. B cell depletion is associated with potent interruption of B-cell trafficking from the periphery to the CNS, decreased presentation of B cell antigens to T cells, modulation of proinflammatory cytokine secretion by B cells, and reduced activation and differentiation of immunoglobulin-secreting plasma blasts. Ocrelizumab is administered every 24 weeks by intravenous infusion. The initial results of the phase 3 trial suggested a low potential risk of increased malignancies, including breast cancer; however, prolonged follow-up showed cancer rates that were consistent with the expected epidemiological rates. Although significant herpes virus infections are now a documented side effect, post-marketing research is typically consistent with clinical trials [63,64]. It is indicated to cure relapsing forms of multiple sclerosis (RMS) and PPMS. According to the label, two 300 mg initial doses are delivered two weeks apart, followed by 600 mg every six months. To prevent infusion reactions, patients should be premedicated 30-60 minutes before ocrelizumab infusion with 100 mg of methylprednisolone and antihistamine. Observe patients for 60 minutes after injection of ocrelizumab injection [62-64].

Rituximab is an anti-CD20 monoclonal antibody that, based on early studies and real-world experience, seems equally effective against RMS and PPMS, although it has never received regulatory clearance [65,66]. Rituximab was approved in 1997 for the cure of lymphoma but is also used off-label for treating several neurological diseases, such as myasthenia gravis and MS. Various dosing regimens have been used. Patients receive 500 or 1000 mg of rituximab intravenously every 6 to 12 months, sometimes after two initial applications conducted two weeks apart [65,66].

Natalizumab is an inhibitor of α4β1 integrin, an adhesion protein produced on the surface of lymphocytes and involved in transmigration via endothelial to the CNS. Compared with placebo or interferon 1a, natalizumab significantly reduces relapses and delays disease progression in patients with RMS advantages retained long-term in real-world investigations [67]. Once every one month, natalizumab is administered as an intravenous infusion [67,68] (Table 2).

**Table 2:** Highly effective disease-modifying therapies for MS ARR: annualized relapse rate, CDP: confirmed disability progression, IFN β−1a: interferon beta 1a, IV: intravenous, SC: subcutaneous, mAb: monoclonal antibody, PPMS: primary progressive multiple sclerosis, RMS: relapsing forms of multiple sclerosis, SPMS: secondary progressive multiple sclerosis. MOA: mechanism of action

Name	Date approved	MOA	Indication	Administration	Efficacy	Side effects	Drug interaction	Reference
Ofatumumab	2020	Anti-CD20 mAb	RMS (first line)	SC injection every four weeks	Reduction in ARR compared with teriflunomide: 54%	Injection reactions, nasopharyngitis, headache, bowel obstruction, and hepatitis	Tofacitinib. Smallpox vaccine. Typhoid vaccine.	[69-71]
Ocrelizumab	2017	Anti-CD20 mAb	RMS and PPMS (first line)	IV infusion every six months	RMS: reduction in ARR compared to IFN βla:47% PPMS: reduction in twelve-week CDP compared to placebo: 24%	Infusion reactions, nasopharyngitis, headache, oral herpes, colitis, hypogammaglobulinemia, neutropenia, and increased cancer risk	Smallpox vaccine. Typhoid vaccine. Influenza vaccine.	[72-75]
Alemtuzumab	2014	Anti-CD52 mAb	RMS (first line)	IV infusion, once daily	Reduction in ARR compared to placebo: 49-69%	Headaches, rash, nausea, pyrexia, thrombocytopenia, hypo- or hyperthyroidism, and encephalitis	Tofacitinib. Siponimod. Ponesimod.	[76-78]
Natalizumab	2004	α4β1 integrin inhibitor	RRMS (second line)	IV infusion every four weeks	Reduction in ARR compared to placebo: 68% Reduction in sustained disease progression compared to placebo: 42%	Fatigue and allergic reaction	Infliximab Tofacitinib.	[79-81]
Mitoxantrone	2000	DNA intercalator	RMS, SPMS (second or third line)	IV infusion every month or three months	Reduction in relapse compared to placebo: 61%	cardiomyopathy, hepatotoxicity, promyelocytic leukemia	Valspodar. Typhoid vaccine. Influenza vaccine.	[82,83]

Dimethyl fumarate is recommended for treating RMS, such as clinical syndrome, relapsing-remitting disease, and secondary progressive disease [7,84]. Dimethyl fumarate is generally well tolerated, but some risk of progressive multifocal leukoencephalopathy [85]. Most of these individuals were lymphopenic; therefore, lymphopenia should be monitored every 6-12 months [84,86]. Fingolimod was the first oral treatment for RMS to be authorized. It prevents lymphocytes from leaving secondary lymphoid organs, thus preventing the infiltration of autoreactive lymphocytes into the CNS [87]. Fingolimod is well tolerated; however, regular laboratory tests have shown mild side effects. Patients having a baseline absolute lymphocyte count (ALC) of 952/ml on the day following the initial dose were higher likely to develop lymphopenia following fingolimod therapy [87,88]. Also, heart block and bradycardia occur when medication is started; therefore, a six-hour observation period is recommended for all individuals receiving their first dose [87,88]. Ozanimod, a newly licensed selective S1P receptor modulator, demonstrated efficacy and safety in RMS [89,90] (Table 3).

**Table 3:** Moderately effective disease-modifying therapies for MS ARR: annualized relapse rate, CDP: confirmed disability progression, CIS: clinically isolated syndrome, RMS: relapsing forms of multiple sclerosis, SPMS: secondary progressive multiple sclerosis, MOA: mechanism of action

Name	Date approved	MOA	Indication	Administration	Efficacy	Side effects	Drug interaction	Reference
Ozanimod	2020	Sphingosine 1-phosphate receptor modulator	CIS, RMS, active SPMS	Oral, once daily	Reduction in ARR compared with placebo: 48%	Headache, hypotension, and herpes zoster	Abiraterone. Duloxetine. Fluconazole.	[89-91]
Siponimod	2019	Sphingosine 1-phosphate receptor modulator	CIS, RMS, active SPMS (first line)	Oral, once daily	Reduction in CDP compared with placebo: 21%	Headache, nasopharyngitis, urinary tract infection, and falls	Alfuzosin. Clozapine. Labetalol.	[92-94]
Cladribine	2019	Not fully known	RMS (second or third line)	Oral, 4-5 days over two-week treatment courses	Reduction in ARR compared with placebo: 55-58%	Headache, lymphocytopenia, nasopharyngitis, neurotoxicity, and nausea	Smallpox vaccine. Typhoid vaccine. Influenza vaccine.	[95-97]
Dimethyl fumarate	2013	Nuclear factor (erythroid-derived 2)- like two pathway inhibitor	RMS (first line)	Oral, twice daily	Reduction in ARR compared to placebo: 48-53%	Flushing, diarrhea, nausea, upper abdominal pain, decreased lymphocyte counts, and elevated liver aminotransferase.	Diroximel fumarate.	[7,98,99]
Fingolimod	2010	Sphingosine- 1-phosphate inhibitor	RMS (second line)	Oral, once daily	Reduction in ARR compared to placebo: 48-60%	Bradycardia, atrioventricular conduction block, macular edema, elevated liver enzyme levels, and mild hypertension	Aripiprazole. Esmolol. Sulpiride.	[87,100,101]

Teriflunomide inhibits dihydroorotate dehydrogenase, an enzyme involved in pyrimidine production. Teriflunomide suppresses the proliferation of lymphocytes considered autoreactive that have been activated [102]. Teriflunomide can treat MS and prevent brain atrophy [102]. Boxed warnings include warnings of hepatotoxicity and teratogenicity. Headache, diarrhea, nausea, alopecia, and a rise in hepatic alanine transferase are typical side reactions. Cholestyramine may be used to quickly remove teriflunomide if necessary [102]. Glatiramer acetate is the acetate salt of a combination of four amino acid-based polypeptides. Its mode of action may include a favorable adjustment of the ratio of proinflammatory to regulatory cytokines [103]. Glatiramer acetate slightly reduces recurrence rates and some disease severity indicators and is considered an equally effective alternative to interferon in RMS [103,104]. Interferon-β moderately decreases the rate of recurrence and MRI disease parameters and delays disability buildup [105]. Interferon-β adverse effects include flu symptoms, subtle laboratory abnormalities, and injection site responses to subcutaneous treatment [105,106] (Table 4).

**Table 4:** Modestly effective disease-modifying therapies for MS ARR: annualized relapse rate, CDP: confirmed disability progression, CIS: clinically isolated syndrome, IFN β−1a: interferon beta 1a, IM: intramuscular, SC: subcutaneous, RMS: relapsing forms of multiple sclerosis, MOA: mechanism of action

Name	Date approved	MOA	Indication	Administration	Efficacy	Side effects	Drug interaction	Reference
Glatiramer Acetate	2015	Not fully known	RMS (first line)	SC injection, once daily or three times weekly	Reduction in ARR compared to placebo: 29%	Injection reactions	Tofacitinib.	[107,108]
PeglFN β-la (Plegridy)	2014	Not fully known	CIS and RMS (first line)	SC, every two weeks	Reduction in ARR compared to placebo: 39%	Injection-site erythema, influenza-like illness, pyrexia, and headache		[109,110]
Teriflunomide	2012	Dihydroorotate dehydrogenase inhibitor	RMS (first line)	Oral, once daily	Reduction in ARR compared to placebo: 32-36%	Nasopharyngitis, headache, diarrhea, and alanine aminotransferase increase	Acyclovir. Methotrexate. Simvastatin.	[102,111,112]
IFN β-la (Rebif)	2002	Not fully known	CIS and RMS (first line)	SC injection, three times weekly	Reduction in ARR compared to placebo: 33%	Injection site inflammation, flu symptoms, rhinitis, and headache	Zidovudine.	[113]
IFN β-la (Avonex)	1996	Not fully known	CIS and RMS (first line)	IM injection, once weekly	Reduction in CDP compared to placebo: 37%	Flu symptoms, muscle aches, asthenia, chills, and fever	Zidovudine.	[105,114]
IFN β-lb (Betaseron)	1993	Not fully known	CIS and RMS (first line)	SC, every other day	Reduction in ARR compared to placebo: 31%	Lymphopenia, hepatitis, and anaphylaxis	-	[115,116]

## Conclusions

MS is a disease of immune-mediated inflammation that attacks myelinated axons in the CNS, causing different degrees of damage. Lidwina of Schiedam recorded the first MS case. After that, Augustus d'Este spent years writing about how his MS symptoms progressed. Currently, its prevalence and incidents are on the rise across the world. Low levels of vitamin D in the blood, genetics, smoking, and infection have been linked to the development of MS. MS patients may be recognized at an earlier and earlier stage of the condition as diagnostic techniques and criteria advance. Furthermore, as a consequence of advances in our knowledge of the pathophysiology and course of MS, extraordinary progress has been made in its treatment. Introducing extremely effective medications has resulted in near-total control of recurring diseases and localized brain inflammation. However, effective progression treatment remains unfulfilled, as existing drugs provide limited protection against the neurodegenerative aspects of MS. MS is often treated with disease-modifying medications such as fingolimod, siponimod, interferon beta, rituximab, natalizumab, and dimethyl fumarate. These medications are effective but have some side effects. Although studies imply that the long-term course of the disease has improved dramatically with the therapy age, more clinical and real-world evaluations are required to obtain evidence of these medications' long-term effectiveness and safety.
